# Supplementary material for: Cytoskeletal Keratins Are Overexpressed in a Zebrafish Model of Idiopathic Scoliosis
Source: Genes (Basel). 2023 May 9;14(5):1058. doi: 10.3390/genes14051058 (PMC10218407; doi:10.3390/genes14051058)
Supplement: Supplementary file 1 [file genes-14-01058-s001.zip › genes-2249768 - Supplemental File S1.pdf]

Supplemental File 1

Melissa Cuevas, Elizabeth Terhune, Cambria Wethey, MkpoutoAbasi James, Rahwa Netsanet, Denisa Grofova, Anna Monley and Nancy Hadley Miller. Cytoskeletal Keratins Are Overexpressed in a Zebrafish Model of Idiopathic Scoliosis.

1) RT-qPCR primer sequences

Supplemental Table 5: Primers used for RT-qPCR experiments

Gene	Primer Name	Sequence 5' -> 3'
KIF7	Kif7 Fwd qRT danio	CAGAGAGCAGATGGATGAGGGTC
	Kif7 Rev qRT danio	TTTGCTGAGGTTGTGGATCAAGC
PTCH1	Ptch1 F Danio	TCA GAG ACT GGC TGC TAG GT
	PTch1 R Danio	ACC GCT GTC TGG TAA TCG TT
GLI1	Gli1 F Danio	TCA GGA GAC GCC TCT TGT TT
	Gli1 R Danio	ACA GAT GCC CGT ATG AGC TG
GLI2	Gli2a F Danio	GGA CCA GCT CGT TCA TCA CAT
	Gli2a R Danio	AAG CCT TCG AGC AAC CTT CA
DLG5	Dlg5 F Danio	CCC TTC CTC CCA TAA CAG CCG
	Dlg5 R Danio	TGC TAC GGG ACG ACT CAG AA
SMO	Smo F Danio	CCT GTG GCG GGT ATC AAC TT
	Smo R Danio	TGC GGT AAA ATT GCC CCT CT
SUFU	Sufu F Danio	GCA AGT CTA TCC TGC CTC CC
	Sufu R2 Danio	ACG ACT CCT TGA TAT CTG CTG G
KRTT1C6 (zgc:77517)	KRTT1C6_qPCR F danio_2	GGAGGAAGGTGTTTGATGCC
	KRTT1C6_qPCR R danio_2	AGACTGCCTGATTGCCATCT
KRTT2C6 (zgc:158846)	KRTT2C6_qPCR F danio	GGCAGATTTACGAGGCGGAAC
	KRTT2C6_qPCR R danio	CTGGCGATTTCCTCATACTGAGC
KRT4	KRT4_qPCR F danio	TGGAAGGAGAGGAGAGCAGAC
	KRT4_qPCR R danio	CTACCACCATAACCAGAGGAAACAC

2.) SHH pathway and *kif7* RT-qPCR results

Supplemental Table 6: RT-qPCR results of wild-type vs *Kif7*$^{co63/co63}$ scoliotic 6wpf, wild-type vs *Kif7*$^{co63/co63}$ non-scoliotic 6wpf, and *Kif7*$^{co63/co63}$ non-scoliotic vs scoliotic 6wpf

<u>AB vs Kif7co63$^{/co63}$ Scoliotic</u>

dCT control	AB GOI - AB ACTB2

dCT experiment	Cur GOI - Cur ACTB2						
ddcT	dCT experiment average - dCT control average						
Fold Change	2^-(ddcT)						
	dCT AB	dCT Scoli	ddCT	ddcT Stdev	Fold Difference	95% Confidence interval (+/-) stdev	
KIF7	9.49	10.84	1.352	0.160	0.392	0.314	0.489
PTCH1	11.62	8.15	-3.474	0.164	11.114	8.849	13.960
GLI1	11.78	10.34	-1.437	0.201	2.707	2.049	3.575
GLI2	10.97	8.57	-2.396	0.791	5.265	1.759	15.756
DLG5	14.10	10.67	-3.438	0.850	10.837	3.335	35.214
SMO	9.81	8.36	-1.449	0.323	2.731	1.745	4.274
SUFU	11.05	9.78	-1.267	1.249	2.407	0.426	13.608
KRTT1C6	5.71	5.80	0.094	0.477	0.937	0.484	1.815
KRTT2C6	6.23	5.61	-0.619	0.196	1.536	1.170	2.016
KRT4	3.96	3.04	-0.927	0.236	1.901	1.371	2.637

AB vs Kif7co63$^{/co63}$ Non-Scoliotic

dCT control	AB GOI - AB ACTB2						
dCT experiment	Non-scoliotic GOI - Non-scoliotic ACTB2						
ddcT	dCT experiment average - dCT control average						
Fold Change	2^-(ddcT)						
	dCT AB	dCT Non-scoliotic	ddCT	ddcT Stdev	Fold Difference	95% Confidence interval (+/-) stdev	
KIF7	9.49	10.86	1.370	0.324	0.387	0.247	0.606
PTCH1	11.62	8.92	-2.699	1.309	6.492	1.058	39.857
GLI1	11.78	11.12	-0.656	1.101	1.575	0.342	7.250
GLI2	10.97	9.23	-1.742	0.758	3.345	1.170	9.562
DLG5	14.10	14.59	0.490	0.815	0.712	0.230	2.202

SMO	9.81	8.51	-1.301	0.515	2.465	1.208	5.031
SUFU	11.05	9.53	-1.519	1.328	2.865	0.455	18.049
KRTT1C6	5.71	5.84	0.128	0.567	0.915	0.417	2.009
KRTT2C6	6.23	5.55	-0.689	0.489	1.612	0.818	3.176
KRT4	3.96	3.42	-0.544	0.900	1.458	0.418	5.076

Kif7co63$^{/co63}$ Non-Scoliotic vs Scoliotic

dCT control	Non-scoliotic GOI - Non-scoliotic ACTB2						
dCT experiment	Scoliotic GOI - Scoliotic ACTB2						
ddcT	dCT experiment average - dCT control average						
Fold Change	2^-(ddcT)						
	dCT Non-scoliotic	dCT Scoliotic	ddCT	ddcT Stdev	Fold Difference	Confidence interval (+/-) stdev	
KIF7	10.86	10.84	-0.017	0.310	1.012	uj	1.557
PTCH1	8.92	8.15	-0.776	1.299	1.712	0.283	10.369
GLI1	11.12	10.34	-0.781	1.085	1.718	0.382	7.731
GLI2	9.23	8.57	-0.654	0.244	1.574	1.123	2.206
DLG5	14.59	10.67	-3.928	0.323	15.225	9.727	23.830
SMO	8.51	8.36	-0.148	0.418	1.108	0.620	1.978
SUFU	9.53	9.78	0.251	0.454	0.840	0.448	1.576
KRTT1C6	5.84	5.80	-0.034	0.460	1.024	0.541	1.937
KRTT2C6	5.55	5.61	0.070	0.463	0.953	0.501	1.811
KRT4	3.42	3.04	-0.383	0.876	1.304	0.387	4.392

Supplemental Table 7 Expression over development Kif7$^{co63/co63}$ vs wild-type, kif7

Formulas	
dCT control	(AB KIF7- AB ACTB2)

dCT experiment	(*kif7*$^{co63/co63}$ KIF7- *kif7*$^{co63/co63}$ ACTB2)
ddcT	(dCT experiment - dCT control)
fold change	2^-(ddCT)

Timepoint	dCT AB	dCT co63	ddCT	ddCT Stdev	Fold Difference	95% Confidence interval (+/-) 2stdev	
D2	9.50	11.29	1.79	0.376	0.290	0.172	0.488
D3	9.78	9.12	-0.66	0.883	1.575348062	0.463	5.354
D4	10.56	11.44	0.88	0.138	0.544624328	0.450	0.660
D5	9.25	6.26	-2.99	0.528	7.961547659	3.828	16.557
D6	9.73	10.57	0.84	0.687	0.560151465	0.216	1.452
D7	10.03	8.40	-1.63	0.490	3.091208247	1.567	6.099
W2	9.49	10.00	0.51	0.855	0.703304924	0.215	2.301
W3	7.52	10.39	2.86	0.235	0.137587322	0.099	0.191
W4	12.00	11.50	-0.50	0.443	1.41490105	0.765	2.616
W5	8.22	10.69	2.47	0.463	0.180352195	0.095	0.343
W6	10.32	11.64	1.31	0.395	0.402390086	0.233	0.696
W7	8.14	9.61	1.47	1.879	0.180352195	0.027	4.881
W8	12.51	12.26	-0.25	0.691	1.187834082	0.456	3.096

Supplemental Table 8: Expression over development wild-type vs 2dpf wild-type, kif7

Formulas	
dCT control	(AB Kif7 D2 - AB ACTB2 D2)
dCT experiment	(AB KIF7 D(n)- AB ACTB2 D(n))
ddcT	(dCT experiment - dCT control)
fold change	2^-(ddCT)

Timepoint	dCT AB D2	dCT AB D(n)	ddCT	ddCT Stdev	Fold Difference	95% Confidence interval (+/-) 2stdev	
D2	9.50	9.50	0.00	0.346	1	1.616	0.619
D3	9.50	9.78	0.28	0.541	0.824579018	1.745	0.390
D4	9.50	10.56	1.06	0.276	0.480638153	0.705	0.328
D5	9.50	9.25	-0.25	0.338	1.189409333	1.900	0.745
D6	9.50	9.73	0.23	0.613	0.852451551	1.993	0.365
D7	9.50	10.03	0.53	0.247	0.694809674	0.979	0.493
W2	9.50	9.49	-0.01	0.655	1.008679277	2.501	0.407
W3	9.50	7.52	-1.98	0.338	3.936029614	6.286	2.465
W4	9.50	12.00	2.50	0.247	0.176970689	0.249	0.126
W5	9.50	8.22	-1.28	0.494	2.428802703	4.817	1.225
W6	9.50	10.32	0.82	0.457	0.566102104	1.066	0.301
W7	9.50	8.14	-1.36	1.843	2.562539985	32.980	0.199
W8	9.50	12.51	3.01	0.376	0.124109869	0.209	0.074

Supplemental Table 9: Expression Over Development Kif7$^{co63/co63}$ vs 2dpf Kif7$^{co63/co63}$, kif7

Formulas	
dCT control	($kif7^{co63/co63}$ KIF7 D2 - $kif7^{co63/co63}$ ACTB2 D2)
dCT experiment	($kif7^{co63/co63}$ KIF7 D(n)- $kif7^{co63/co63}$ ACTB2 D(n))
ddcT	(dCT experiment - dCT control)
fold change	2^-(ddCT)

Timepoint	dCT kif7co63 D2	dCT kif7co63 D(n)	ddCT	ddCT Stdev	Fold Difference	95% Confidence interval (+/-) 2stdev	
D2	11.29	11.29	0.00	0.404	1	1.751	0.571
D3	11.29	9.12	-2.17	0.793	4.486895233	13.463	1.495
D4	11.29	11.44	0.15	0.290	0.904174815	1.352	0.605
D5	11.29	6.26	-5.03	0.554	32.70890216	70.456	15.185
D6	11.29	10.57	-0.72	0.488	1.649348036	3.245	0.838
D7	11.29	8.40	-2.89	0.566	7.418758712	16.270	3.383
W2	11.29	10.00	-1.29	0.666	2.450382504	6.168	0.973
W3	11.29	10.39	-0.90	0.288	1.870569724	2.789	1.255
W4	11.29	11.50	0.21	0.526	0.86489729	1.794	0.417
W5	11.29	10.69	-0.60	0.335	1.51304135	2.408	0.951
W6	11.29	11.64	0.35	0.298	0.786826843	1.190	0.520
W7	11.29	9.61	-1.67	0.526	3.192955101	6.617	1.541
W8	11.29	12.26	0.97	0.691	0.509212699	1.328	0.195

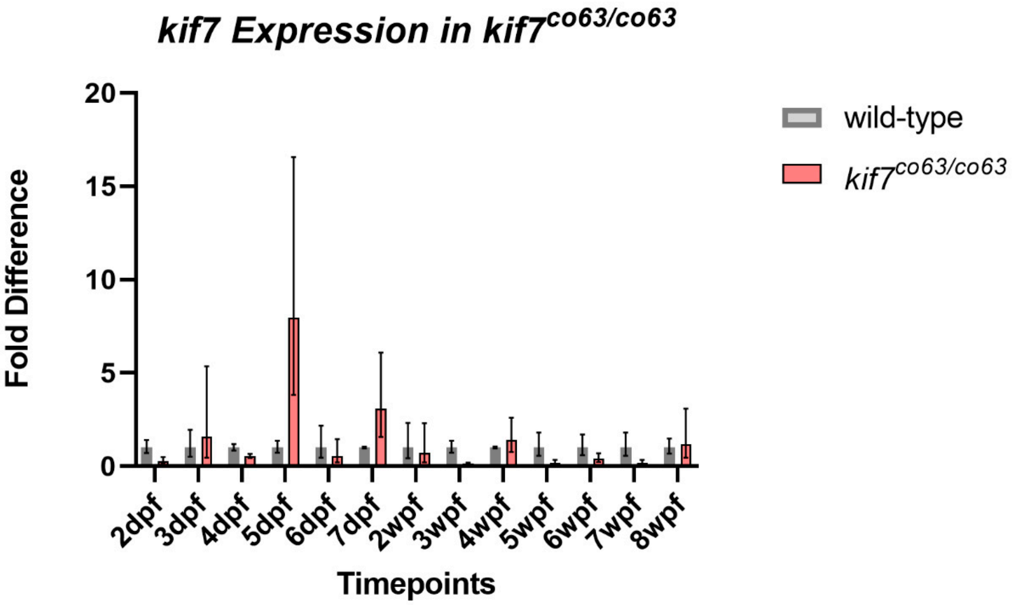

Supplemental Figure 1: *kif7* expression over zebrafish development in $kif7^{co63/co63}$ as compared to wild-type at each corresponding timepoint. Wild-type was used as the comparison sample, so was set at a fold change of “1” for each timepoint.

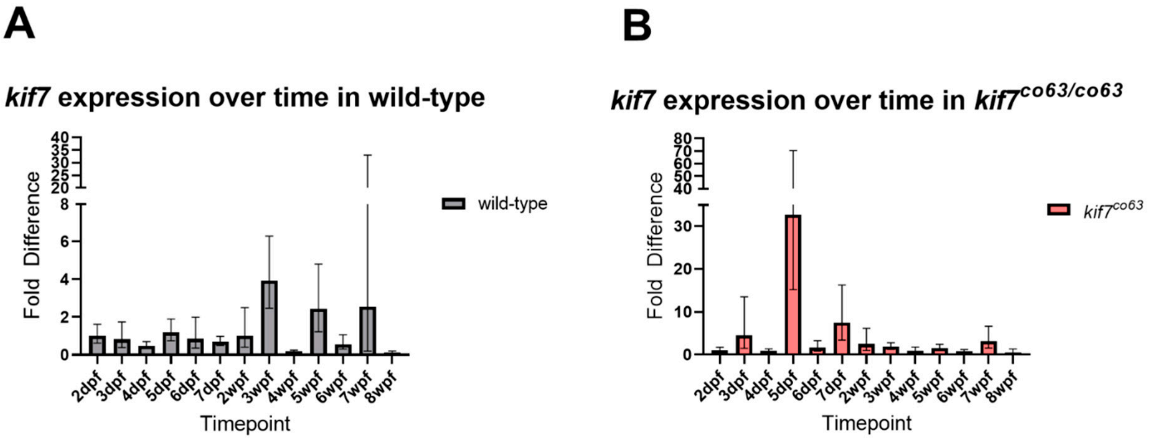

Supplemental Figure 2: Comparison in *kif7* expression over time in wild-type (A) and in *kif7*$^{co63/co63}$ (B) starting with 2dpf. Expression of *kif7* in wild-type is seen to be consistent during early development with higher expression seen closer to juvenile age. *Kif7* expression in *kif7*$^{co63/co63}$ has the opposite trend with higher expression observed earlier in development.

3.) Additional RNA sequencing figures

Supplemental Figure 3

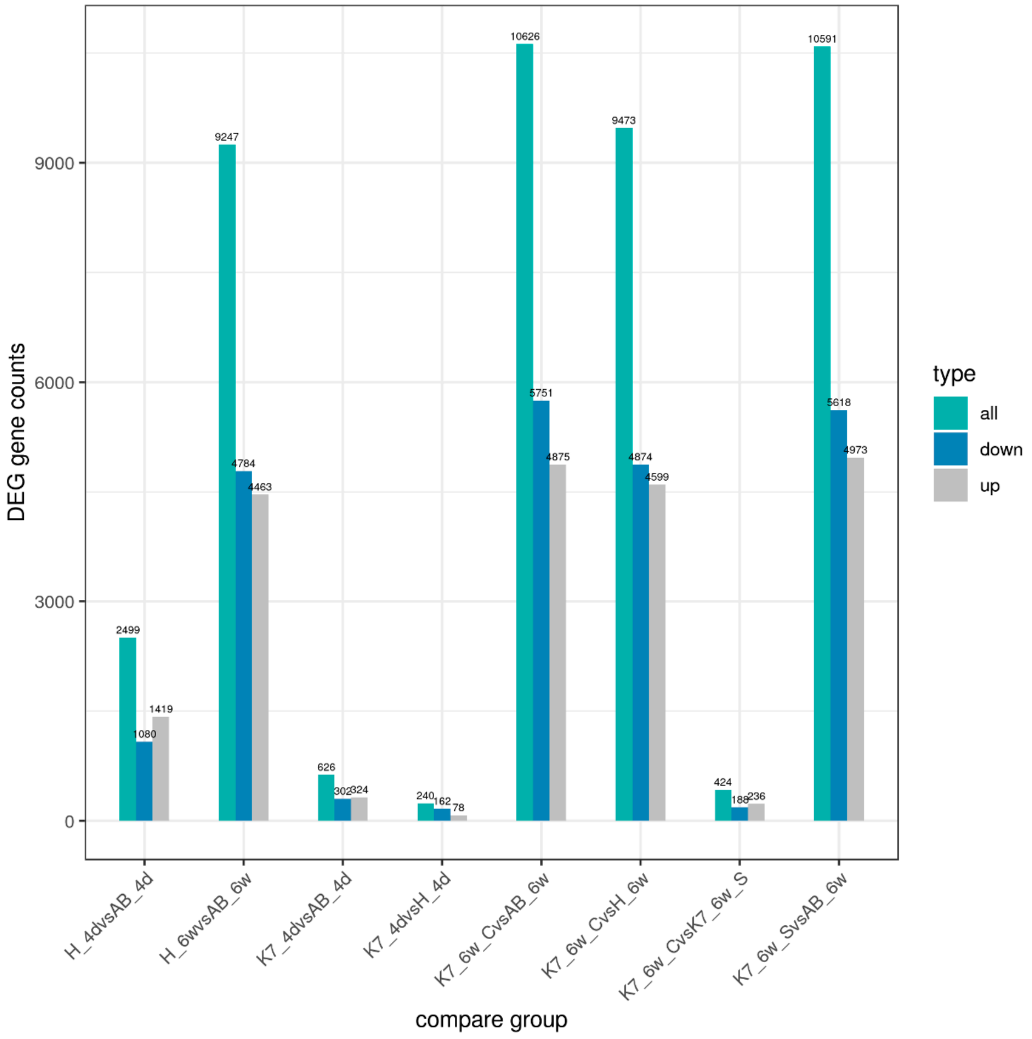

Supplemental Figure 4

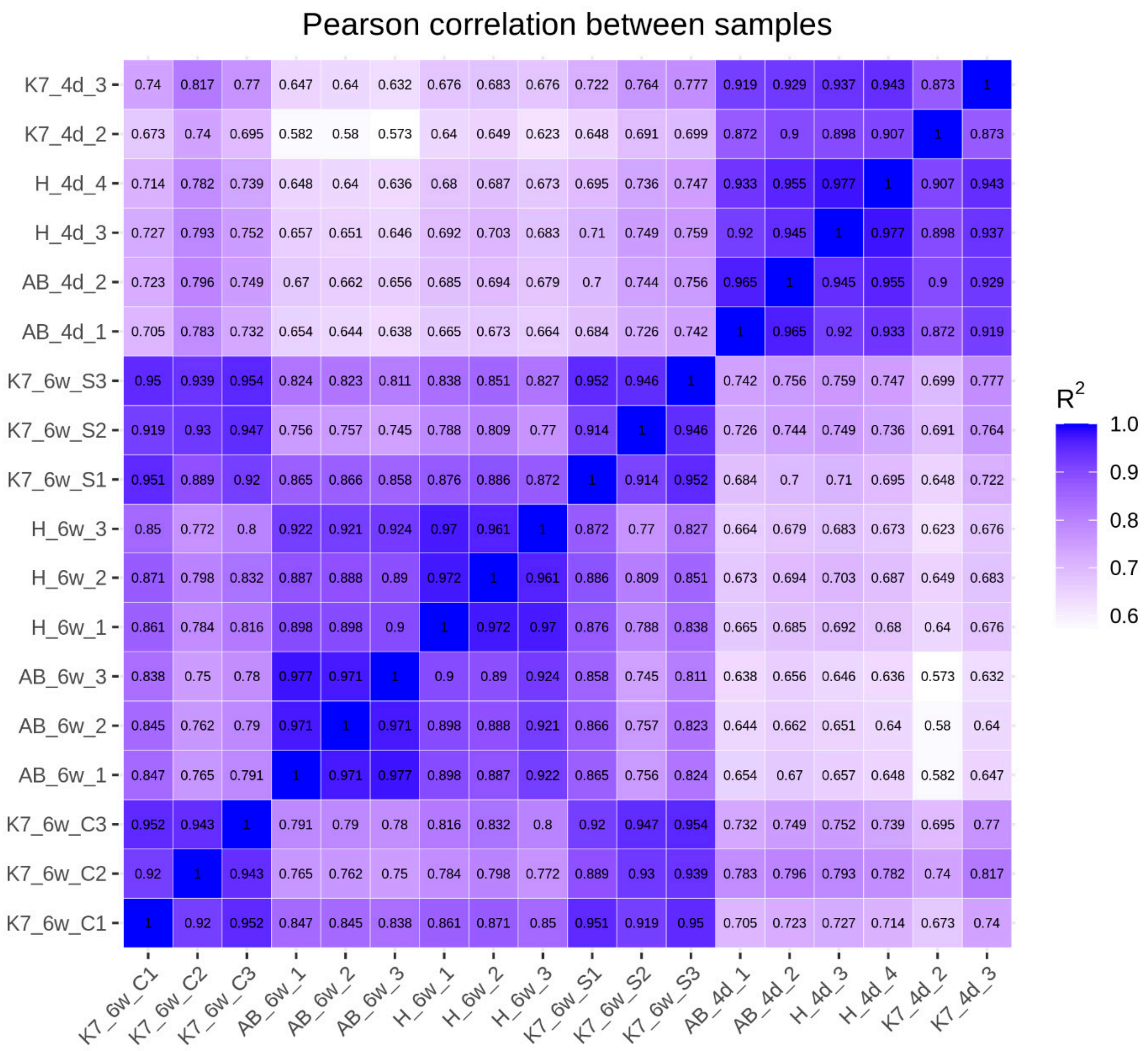

4) Genotyping gel of *kif7*$^{co63/co63}$

Cuevas et al., Supplemental File 1

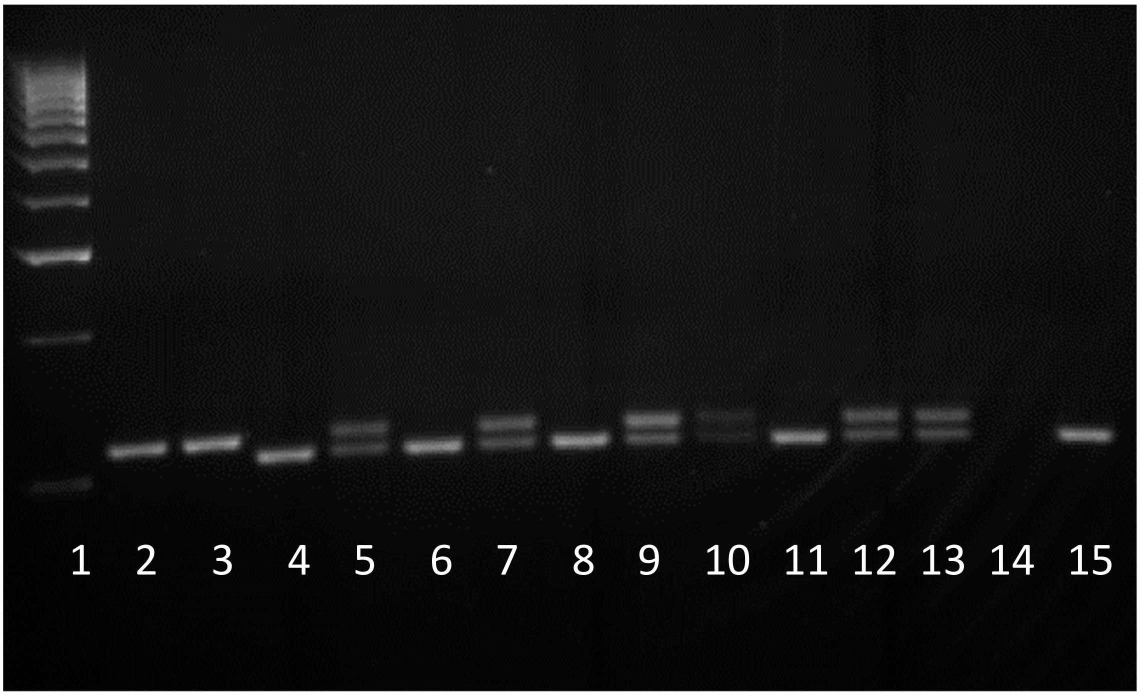

Supplemental Figure 5: Agarose gel depicting wild-type (lanes 4,6,8,11,15) , kif7$^{+/co63}$ (lanes 5,7,9,10,12,13) and kif7$^{co63/co63}$ (lanes 1,2). Negative control: lane 14. Positive wild-type control: lane 15

5) Complementation test of two *kif7* alleles

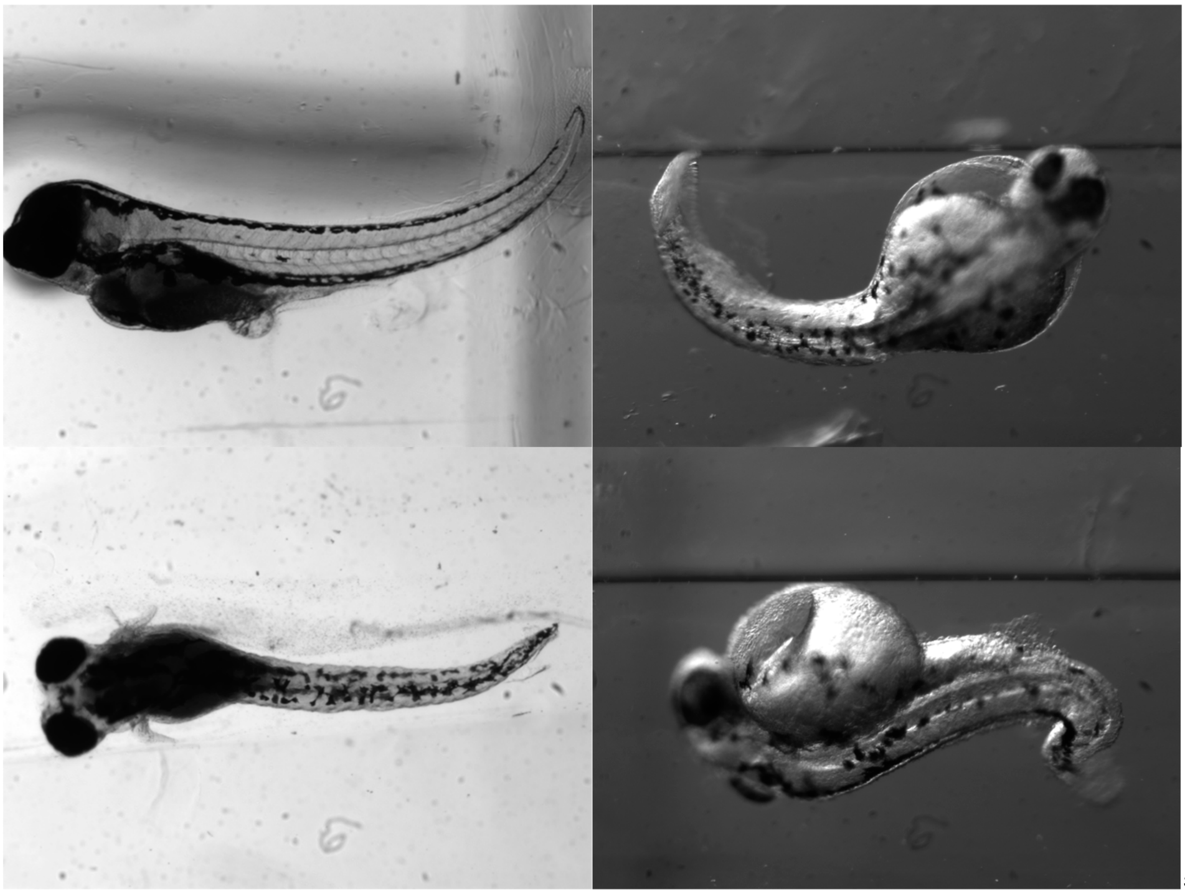

5)

Supplemental Figure 6: complementation test of *kif7* $^{co63/co63}$ female X kif7 4bp deletion male and *kif7* $^{co63/co63}$ male X *kif7* 4bp deletion female yielded fish with similar death rates and offsprings with spinal malformations. 34% survived and matured as expected at 24 hpf. ~46% of embryos died from cross. ~20% of embryos were underdeveloped at 24hpf.
